# Exoplanets, extraterrestrial life and beyond: an interview with Douglas Lin

**DOI:** 10.1093/nsr/nwac008

**Published:** 2022-01-17

**Authors:** Ling Xin (辛玲)

**Affiliations:** Science writer based, Beijing

## Abstract

In a survey released by the US National Academies of Sciences, Engineering and Medicine in November 2021, the search for Earth-like planets outside our solar system was identified as a major goal for astronomy over the next 10 years. National Science Review invites Douglas N.C. Lin (林潮), emeritus professor of astrophysics from the University of California, Santa Cruz, who has studied planet formation, evolution and solar system dynamics for almost five decades, to look back on exoplanet research, a field that has expanded exponentially in the past 30 years, and to envision the opportunities and challenges in our search for habitable worlds. As the founding director of the Kavli Institute for Astronomy and Astrophysics at Peking University, Lin offers his insights on how China's astronomical community can benefit more from collaboration, and what young scientists could do to seize the opportunities offered by what he calls ‘a golden era of exoplanet research’.

## THE SEARCH FOR EXOPLANETS

***NSR***: The Astro2020 Decadal Survey listed exoplanet research as a top priority. Why now?

***Lin***: The field of exoplanet research is blossoming after three decades of exponential growth. First, we now have a technical window of opportunity, as various instruments used to look for exoplanets from space, such as the Kepler space telescope and the Transiting Exoplanet Survey Satellite (TESS), were built after discoveries made by telescopes built on the ground, and it took a few years for them to come into full production. Second, the exoplanet community has been built up. Thirty years ago, only a handful of people were working in the field, not knowing whether it was going to lead anywhere. Now the community has expanded tremendously. Third, the concept of what to do next has been well thought out. These are well-defined big questions such as the origin of life and life elsewhere in the universe. There is a huge opportunity for us to answer these questions with an interdisciplinary approach, by combining Earth and planetary science, astronomy, biology, chemistry and physics using ground-based and space-borne telescopes.

***NSR***: When human beings first thought about other planets, were they driven by the question ‘are we alone in the universe’?

***Lin***: I think the word ‘we’ in this question refers to both the solar system and life. It is interesting that the notion of many coexisting planetary systems was already conceptualized by the Chinese poet Qu Yuan and postulated by the Greek philosopher Epicurus over 2000 years ago. Then, more than 200 years ago, the French mathematician Pierre-Simon Laplace constructed the first quantitative model about planet formation, and he suggested that the planets in our solar system had coalesced from dispersed materials in a disk orbiting the Sun. There were also alternative scenarios, proposed mostly by theorists, about how planets may have formed and evolved.

**Figure:**
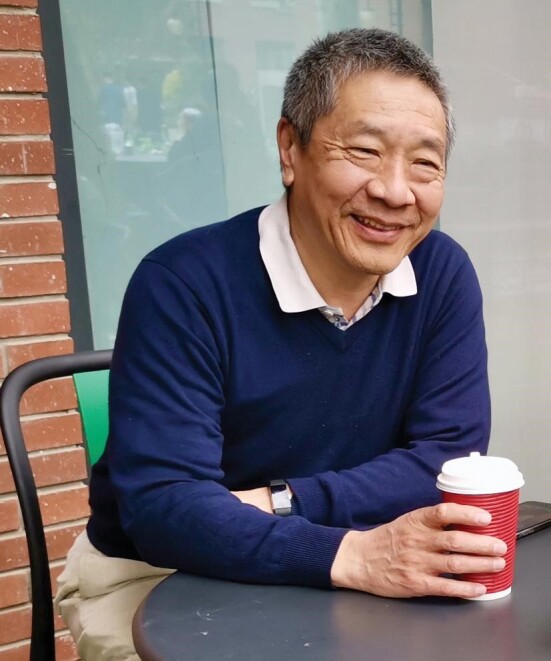
Douglas Lin at a coffee shop conversation on the Tsinghua University campus in 2018 *(courtesy of Douglas Lin).*

In the 1970s, humans finally had the capability to send probes to image landscapes on other planets in our solar system, so we started to see their structures, which are very different from our own. Then, in the early 1990s, infrared satellites were launched to detect protoplanetary disks around young stars. It was finally possible to understand how planets may have formed in active settings similar to those Laplace had envisioned. However, painstaking searches for bona fide planets outside the solar system endured many failed attempts and some false alarms. Early ‘planet hunters’ almost gave up their quests at various points. I remember going to a conference on ‘planetary systems formation, evolution and detection’ in Hawaii in 1994 and several people who had been methodically looking for Jupiter-like planets were converging on the disappointing conclusion that such planets may be very rare and very hard to find.

***NSR***: How did you get into the field in the first place?

***Lin***: By accident. When I was working at Cambridge University as a PhD student in the mid-1970s, I applied accretion disk modeling to the study of some recently discovered binary stars that are powerful sources of X-ray emission. They were thought to be composed of dense neutron stars or black holes collecting gas from their nearby Sun-like normal stellar companions, and I was carrying out computer simulations on the transfer of mass between these stars.

One night when I was working in the university computer lab at 4 am, I made a programming mistake in the specification of my input parameters. Instead of giving a companion star a fraction of the Sun's mass as I had initially planned, I accidentally specified its mass to be comparable to that of Jupiter. The next day when I went to pick up the output of my simulation, I saw a beautiful spiral pattern, which I was not expecting at all.

In a series of follow-up analyses, I worked with a colleague to show that the spiral pattern actually indicated a strong tidal interaction between Jupiter and its natal disk, and this effect can lead to changes in Jupiter's orbits. So, during the 1994 meeting I emphatically claimed, ‘It's not that Jupiter-like planets rarely form; they do form prolifically, but some, perhaps most, of them may have moved into their host stars’. Nearly all the participants at the conference thought I was crazy.

***NSR***: But scientists soon discovered 51 Pegasi b, the first exoplanet known to orbit a Sun-like star. What surprised you most about 51 Peg b?

***Lin***: Ironically, around the same time as the Hawaii conference, 51 Pegasi b was found by two Swiss colleagues at the Haute-Provence Observatory in France. After its announcement, this discovery was quickly confirmed by astronomers in California. The big surprise to nearly everyone, including the discoverers, was that it only takes this planet about four days to complete an orbit around its host star, in contrast to the 12-year orbit of Jupiter around the Sun. This difference implies that 51 Pegasi b's distance from its host star is 100 times smaller than that between Jupiter and the Sun. At such a close range, it is difficult to form planets along the lines of conventional models.

In my work with a colleague 10 years earlier, we did anticipate that infant planets would migrate through their natal disks, but I did not expect them to stop near the star. I immediately looked into how planets can be relocated and preserved in the proximity of their host stars. Within a few hours, I came up with the innovative idea that their migration may be stalled when planets enter a central cavity in the disk, which is carved out by their host star's strong magnetic fields. I also suggested that the tidal force between stars and very close planets may be able to halt their migration. Later, other ideas were proposed to explain why planets stop after migration. But my proposition has been widely accepted as the most likely scenario for the origin of these close-in planets, especially those in multiple-planet systems.

**Figure:**
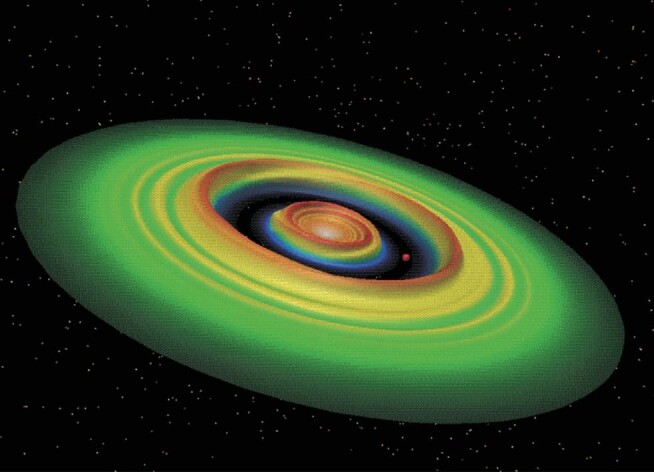
A computer simulation of an emerging Jupiter-like gas giant planet in its natal protostellar disk. This theoretical prediction was confirmed by subsequent observations with the ALMA telescope *(courtesy of Douglas Lin and Geoff Bryden)*.

***NSR***: Following the discovery of 51 Peg b and other exoplanets, what are the most important changes of paradigm in our concept?

***Lin***: The first major paradigm shift was the realization of the ubiquity and diversity of planets. Now we know that planets exist around almost every star, and their characteristics are widely diverse. The second conceptual leap forward is the appreciation of planets with astonishing mobility throughout their lifespan, from infancy to the demise of their host stars. The diverse kinematic architecture of exoplanetary systems has inspired us to examine the issue of dynamical stability and evolution, as their affiliates migrate over extended distances due to different contributing effects. Their mobility and diversity have lots of potential implications for the origin of life and the prospect of biological evolution in exoplanetary systems. Transformation of the physical conditions of planets is likely to be a driving force for both mutation and natural selection. Although changes in the surface conditions on Earth have led to transitional bursts of biological diversification and proliferation as well as mass extinction, these variations are minor in comparison with those in systems where planets move all over the place and frequently go through collisions much more powerful than the one that may have wiped out the dinosaurs on Earth. What is the probability of life emerging, thriving, dwindling and surviving? Well, we do not have an answer yet. But attempts to understand the kinematics and structure of exoplanets will open up our minds with regard to the realm of feasibilities.

***NSR***: We have found more than 4500 exoplanets so far. What techniques do we use to spot them?

***Lin***: There are many ways to look for planets out there. The most straightforward way is the direct imaging method. You just look up into the sky at the right place and right time, as ancient Greek and Chinese astronomers did when they spotted the planets in our solar system. Since they move relative to the Sun and background stars, our ancestral observers named them planets, ‘wanderers in the sky’, or *xingxing* (行星), ‘traveling stars’. It is difficult to see Mars, Jupiter and Saturn during the day because the Sun's light is too bright. Similarly, from our vantage point, exoplanets are very close to their host star, which usually outshines them billions of times over. The technical challenge with this approach is the attempt to effectively block the star's light so that the reflected light from the planets can peek through.

A second campaign to search for planets is the radial velocity survey. Due to mutual gravitational attraction, planets and their host star perturb each other's motion. If there are some slight but periodic changes in the star's spectral lines, there is a good chance that these signals are triggered by the gravity of some orbiting planets. That was how our Swiss colleagues first discovered 51 Peg b and observers from many other countries, including China, subsequently found many hundreds more.

The third technique is the transit method. The transit phenomenon is similar to a solar eclipse. When a planet goes between us and its host star, it blocks out a little bit of the star's light. It is technically demanding to precisely measure the minute changes in the stellar light, but this procedure happens to be the most efficient way of identifying several thousand exoplanets, including those by NASA’s Kepler and TESS missions. It also has value-added advantages in collecting candidates for follow-up characterization of the chemical composition and physical make-up of the planets’ atmospheres during their transits. In fact, this tactic is similar to watching the hair of rock stars in silhouette when they perform on stage with some spotlights behind them.

I think the beauty of this field is that you have multiple complementary techniques to characterize different aspects of a planet, and no single technique is wholly superior to the others.

—Douglas Lin

Besides the methods mentioned above, there are several other ways, including astrometry (measuring the position changes of host stars), gravitational microlensing events (monitoring characteristic brightness change due to the bending of the light path by a planet's gravity) and using radio signals from the host star to infer the existence of nearby planets. Several dozen planets have already been discovered with the microlensing method and it has the exciting prospect of detecting planets with mass and orbits comparable to those of the Earth.

I think the beauty of this field is that you have multiple complementary techniques to characterize different aspects of a planet, and no single technique is wholly superior to the others. You can do sophisticated measurements with large telescopes like Keck, but scientists also used a four-inch-aperture telescope to make the first detection with the transit method from a parking lot in Denver, Colorado. When I first heard about that, I thought, ‘Wow, this is going to go far!’ And it did.

## LIFE IN OTHER WORLDS

***NSR***: How far have we come with regard to the search for life beyond Earth?

***Lin***: Although we have made lots of progress in understanding the physical properties of planets over the past 50 years, our ideas on the origin of life remain extremely primitive and speculative because there is not yet any experimental data to tell us definitively one way or the other.

When I gave public talks, I liked to ask my audience two questions. The first question was, ‘Who believes there is life beyond Earth?’ About 90% of the people would raise their hands. Then I asked, ‘Who knows if there is firm evidence of life beyond Earth?’ Nobody raised their hand. So everybody has an opinion but nobody has any well-established facts.

That is why we need to keep on searching for clues and constraints. Our detection and characterization abilities are still very limited and our theoretical constructs are very preliminary. Nevertheless, persistent effort will gradually enable us to improve our perception and ability to comprehend the big picture.

***NSR***: When we look for life on other worlds, what are the biosignatures to identify?

***Lin***: There is no shortage of the most common elements in known living organisms, such as carbon, hydrogen, nitrogen and oxygen, on exoplanets. By the most basic standards, there is even no lack of known habitable planets. However, there is a scarcity of easily detectable signs of life.

It may be more fruitful to first look for circumstantial indicators of living species. For instance, when you go to the Tibetan Plateau to spot snow leopards, it is much easier to sift through their footprints over extensive rugged terrains. On Earth, the most easily identifiable ‘footprint of life’ is the vast amount of molecular oxygen in the atmosphere. These molecules are *not* the necessary ingredients, but waste byproducts, of microbial life. Biological processes dramatically enriched the molecular oxygen content of Earth's atmosphere during the Archean Eon. If we completely sterilized all life forms on Earth today, the molecular oxygen in the Earth's atmosphere would quickly plummet because oxygen is very reactive with other elements. A good example is Mars, where the oxygen rusted the iron on the surface to make its top soil appear reddish.

Astronomers choose molecular oxygen as a biomarker because it is relatively easy to detect. Oxygen is the third most abundant element in the universe, though it does not commonly exist as free molecules in the atmosphere of most stars and planets. Traces of oxygen molecules are not difficult to pick up from the infrared waveband. They are even possible to detect in visible light if they exist in the form of ozone. Most astronomers believe that if we detect a high concentration of molecular oxygen in the atmosphere of a planet, there is a reasonable chance it is an indicator of carbon-based life on the surface of that planet. Meanwhile, if a planet has an ozone layer in its atmosphere, we may even be able to detect the traces with ground-based telescopes.

Just last year, the identification of a trace of phosphine on Venus was made. Since phosphine is primarily produced by living organisms on Earth, it has been interpreted as a biomarker, a likely sign of life among the high clouds of the extremely hot Venus atmosphere. This proposition received a chorus of healthy skepticism with regard to whether the spectroscopic fingerprints may be masked by sulfur dioxide at the same wavelength. Some scientists refuted the marginal signals altogether based on their own observations and analyses. Such debates are normal in the scientific community. As the astrobiology pioneer, Carl Sagan, succinctly put it: ‘Extraordinary claims require extraordinary evidence.’

***NSR***: If we ever detect molecular oxygen on exoplanets, what would that mean?

***Lin***: It will be just the first step. Rich supplies of oxygen can also come from other sources such as photo-dissociated water molecules. It is important to distinguish between oxygen molecules related to biological activities and those from other sources. The scenario is extremely complicated because it involves cross-disciplinary interpretation of competing biological, chemical, physical, geological and atmospheric processes.

Besides molecular oxygen, we need to observe other biosignatures as well. Even with the most powerful ground-based and space-borne telescopes, state-of-the-art instruments and software, it will take time to observe biosignatures on a handful of carefully selected habitable planets on a one-by-one basis. Maybe 10 years from now, we will have sufficient data to say that potential biosignatures have been found in either 10%, 1% or 0.1% of all the planets we have examined. We can then go on to compare the newly found biomarkers’ dependence on the location of planets, the age and composition of their host stars, etc. with respect to the habitable zones. In this pursuit, both the positive detection and stringent upper limits of biosignatures will have profound implications on the ubiquity of life, at least in the microbial form, elsewhere in the universe.

**Figure:**
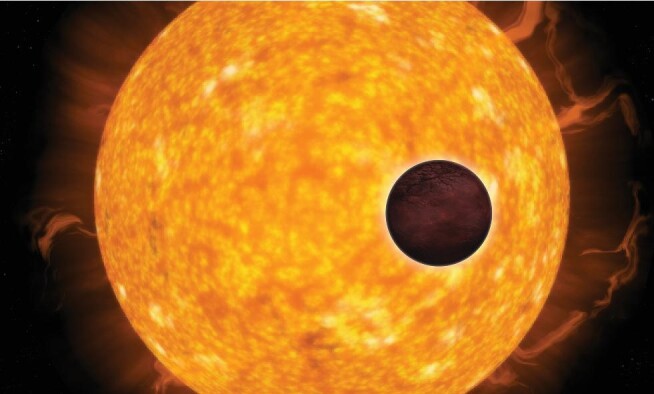
A small, rocky planet transiting in front of its host star. The partial blockage of the stellar light enables astronomers to search for traces of biomarkers in the planet's atmosphere *(credit: ESA).*

***NSR***: If extraterrestrial life was quite different from the life forms on Earth, if it didn’t need water or wasn’t carbon-based, how are we supposed to find it?

***Lin***: Before we start to search blindly for signs of other forms of life, we need to reach a consensus on a clearly stated definition of ‘life’. A frequently used standard is ‘creatures capable of self-assembly and self-reproduction’. With that interpretation, we are on the verge of being able to manufacture the first silicon-based life in the form of smart robots with biomarkers completely different from those of carbon-based life. Besides the technical challenges of remotely detecting either the traces or footprints of such entities, their very qualification as life forms remains highly controversial.

Former US Defense Secretary, Donald Rumsfeld, once separated the ‘known unknowns’—things we know that we don’t know, from the ‘unknown unknowns’—things we don’t even know that we don’t know. This question falls under the ‘unknown unknowns’ category.

I think we probably need to start with the known unknowns and carry out the search for life step by step. It is always easier to first look for life forms like those on Earth because we know what to look for. I admit this approach is geocentric and narrowly focused. But if we don’t have any searching criteria based on prior knowledge, I’m afraid we will not even know which questions to ask.

***NSR***: What should we do if we do find extraterrestrial life someday?

***Lin***: My inclination is to do nothing until we first carefully observe them and gain a deep understanding of their characteristics, behavior, superiority and vulnerability. In general, it is technically difficult for a biological species to survive a long and arduous journey between their home base and Earth in a life-supporting, artificial or natural enclosure. It is prudent and wise to be cautious, especially when we have no idea if that life form may be welcoming, friendly, xenophobic, defensive or antagonistic in nature.

Any realization that there is evidence of microbial life out there is already good enough to enrich our perception and understanding of the routes to biology on initially barren but potentially habitable planets. This knowledge will also open a window of opportunity to break down cross-disciplinary boundaries and to develop a holistic approach in our attempts to understand nature.

Anyway, my advice is don’t worry and be happy! I prefer to objectively make sense of the world around us than to overreact due to lack of empathy, or prejudicially latch on to ignorance derived from some preconceived convictions.

***NSR***: How does the search for exoplanets and life help us understand earthly problems, such as climate change?

***Lin***: If we look at Venus and Mars, we can already anticipate some consequences of global climate changes. It was a lot warmer here on Earth during the Cretaceous period, but the climate transitions in geological history happened over a really long period of time. What is alarming about today is the astonishing rate at which carbon dioxide and other greenhouse gases accumulate in the atmosphere, largely due to human factors. Climatic models have predicted major changes in weather patterns and coastal boundaries within our children's generation. There is a sense of urgency to quell this trend.

Although current political discussions by world leaders have mostly focused on cutting carbon emissions, it is also important to explore carbon capture technologies. We can get some inspiration in this regard by understanding atmospheric circulation and climatic dynamics on other planets. For instance, the greenhouse effect on Venus is so strong that rain evaporates before it reaches the ground. We do not want this to happen on Earth, because it would cut off a major channel of carbon return from the Earth's atmosphere to the soil and rocks on Earth's surface. We need to unravel the complex interplay between air, ocean, sea ice, land surface, atmosphere chemistry and biological and industrial activities on a global scale as well as to understand the long-term carbon deposit-and-release geophysical cycle in and beneath the Earth's rocky crust. This comprehensive knowledge will be useful for strategizing effective, holistic approaches to quench and hopefully to thwart the current trend of rapid global warming.

***NSR***: What are your hopes for the search for life in the future?

***Lin***: A major challenge is to find evidence of life beyond any reasonable doubt. Let's think beyond the exhaustively discussed issues of habitable zone and what life may look like under extreme environments. There are many other fascinating enigmas for us to contemplate, including the paradox of why microbial life emerged so quickly on Earth, almost as fast as it was possible—while there is no conspicuous sign of life on the surface of Mars today, even though it was once widely covered with liquid water and blanketed by an atmosphere similar to the one on Earth when life started here. The issue of whether microbial life ever emerged on Mars remains a mystery. If so, what did it take for the basic information of life to get started there and how did life become essentially extinct on its surface? No one really knows the answers, or even how to construct a hypothesis that is testable at the moment.

Nevertheless, we have good reasons to be optimistic about the future. Just four years ago, we detected the first interstellar object, Oumuamua, passing through our solar system. A vast population of similar objects in interstellar space have been extrapolated for their sighting probability. If they are as bountiful as we anticipate, we will be able to identify hundreds of them per year with the Vera Rubin Observatory, which will come online soon. Presumably, these objects began their space odyssey and carried with them information from the planetary neighborhood around some other stars. Meanwhile, we have already refined the technique of extracting samples from asteroids, comets and moons of planets in the solar system. With these capabilities, we will be able to trace the past trajectories of those interstellar objects and analyze the composition of returned samples. These data will greatly augment valuable clues as to the conditions of faraway planetary systems, stimulate new ideas and constrain contrastive hypotheses.

## CHINA’S OPPORTUNITIES AND CHALLENGES

***NSR***: What are China's efforts in the search for exoplanets?

***Lin***: There are at least three areas where Chinese scientists are already making progress. First, they have been using the transit method with telescopes in the South Pole and other places to discover a number of exoplanet candidates. Some young scientists recently returned to China have used the Large Sky Area Multi-Object Fiber Spectroscopic Telescope, which is not far from Beijing, to measure the orbital eccentricity and characterize the host stars of some transiting planets previously detected by Kepler. Their study excavated important clues as to the conditions for the prolific emergence of super Earths. Meanwhile, faculty and students at Tsinghua University are actively involved in the microlensing method for exoplanet detection, primarily through a collaboration with a group of observers from South Korea. Their campaign has unveiled a rich class of Neptune-like planets, which are below the detection limits of all previous exoplanet searches with other approaches.

For the near future, the Shanghai Astronomical Observatory is leading the development of a space mission called the Earth 2.0 Transit Planet Survey. Otherwise, as far as I know, the National Space Science Center under the Chinese Academy of Sciences is contemplating an astrometric mission to look for terrestrial exoplanets, and the Five-hundred-meter Aperture Spherical radio Telescope (FAST) based in Guizhou, plans to assign some observing time to detecting radio signals associated with exoplanets.

Last but not least, China, along with the USA, Japan, Canada and India, is a founding member of the Thirty Meter Telescope (TMT) project, a next-generation telescope set to characterize the atmospheres of some transiting exoplanets. The new *Decadal Survey* has recommended it, together with the Giant Magellan Telescopes, as the top funding priority for the US National Science Foundation, among ground-based astronomy facilities. Whether TMT China will survive the ongoing political tensions between the USA and China remains to be seen, but I do hope projects like this will become bridges to connect countries and benefit scientific communities on all sides. In an ideal world, fundamental science has no national borders or subject divisions. I hope all these efforts will lead to very positive results.

***NSR***: Does China have any unique advantages in exoplanet research?

***Lin***: Yes. China has a logistic edge in long-term ground-based transit follow-up observations, because the targeted stars need to be continuously monitored from different longitudes around the Earth, and the coverage over the western regions of China, including Tibet Autonomous Region and Xinjiang Uyghur Autonomous Region, has been almost non-existent. There are many things China can do to make up for this geographical gap. The FAST telescope has the world's highest sensitivity when it comes to radio signal detection.

The Chinese astronomical community also has some disadvantages. In order to detect planets that may host life, we do need to observe in the infrared wavelength, and China's infrared detector technologies have been lagging behind. Some of those devices have been on the US government's embargo list for China for many years. China needs to decide, at some point, either to settle and use something less than state-of-the-art, or to develop her own expertise. The latter commitment will require resources and talents, and it will take time to accomplish.

China has a rapidly growing space program primarily centered around solar system exploration, including missions to the Moon and Mars. Some groups are considering the possibility of collecting rocks from asteroids and comets. These samples are the oldest relics in the solar system and they will provide pivotal clues on its formation and early evolution. China is also developing a space telescope in conjunction with its space station. Such facilities will be essential for the characterization of exoplanets’ atmospheres.

One major limitation with China is that there are not enough experts in the field.

—Douglas Lin

***NSR***: Do you think the Chinese community is ready to take on opportunities offered by this round of technological advancements?

***Lin***: The community is definitely growing. One major limitation with China is that there are not enough experts in the field. I have been pushing forward exoplanet research in China for the past 15 years. I talked with planning and funding agencies, but they said there were not enough active participants in the field. It is natural for any community to have a preference for supporting prevailing areas that are better established. However, supports for emerging frontiers, including exoplanet research, are likely to be pivotal precisely because there are not yet enough pioneers. Ironically, many researchers in Europe and the USA were students or postdoctoral fellows in their twenties at the beginning of the planetary astrophysics surge, and they have matured into the backbone, the movers and shakers of today's fastest growing branch of astronomy.

But it is never too late to catch up. Many Chinese students who went to graduate schools in the USA and Europe were actively engaged in exoplanet studies. Some of them have now returned to China with expertise on both the observational and theoretical sides. These promising ‘new bloods’ have the

potential to lead major frontier projects in China. In addition to some of the projects I have mentioned, there are many initiatives now being proposed or well underway. I hope these young scientists will also improve the culture of how science is done in China, and bring a breath of fresh air into all aspects of scientific enterprise, including strategic planning, genuine collaboration in research and vibrant international collaboration.

***NSR***: Big science projects require collaboration. How can the astronomy community in China work together more effectively?

***Lin***: This is a genuine challenge. China may have one of the most competitive societies in the world. Children are being evaluated and compared shortly after they are born. Capability and performance assessments are regularly administered through all kinds of grades and exams from elementary school to college. Although quantitative appraisals and standardized judgements ensure fairness, stimulate accomplishments and promote pride, an intense drive and thrilling race may also lead to invisible barriers to individuals joining forces. In academia, contributions of researchers are often judged and rewarded based on the impacts of their publications. The simplest metrics to use are the ranking order of authorship, citation counts, grants and prizes. Unfortunately, a single paper can only have one first author with one leading institution. A mechanical formula may be a straightforward accounting measure for the promotion of individuals and their host institutions, but awkward negotiations over the leadership may also be detrimental for long-term, multi-team collaborations.

Let's use Switzerland as an example. The country has four national languages and a very diverse culture, but it has been very successful in many scientific and technological fields, especially in the area of exoplanet research. How could that be? I think there is a lesson for the Chinese community to learn. One thing all scientists do is compete for research resources. However, after a decision is made about a major project, what the Swiss are able to do is get the losing side to join the winning side, and they push the project forward together. However, very often in China, I see losing teams sit on the side and watch the winning team succeed—or fail. The goals of the scientific community should override an individual institution's interests, and consensus-building and team cooperation should take priority over personal glory, primacy and authority.

Collaboration is particularly important in astronomy because modern observers look over a wide range of wavelengths and they need to exchange information to get a complete picture of a given phenomenon. Also, one characteristic of astronomy is that, with the expeditious advancements in technologies, its major research direction changes about every decade or so. It is really important to reach a general accord among scientists as to what will be the nationwide priorities in the short, medium and long-term future. The process of consensus-building needs thorough discussions, critical evaluations, inclusive debates and a global vision. The *Decadal Survey* we mentioned was produced through such a process. These studies look beyond the interest of any individual institute or sub-discipline, and they reflect the spirit of community collaboration.

The astronomy community in China is in need of reform towards a fully functioning decision-making mechanism. Although there are many widely represented panels to discuss future plans, decisions made by some committees at one level are overturned by other committees at another level, or by other groups with vested institutional or sub-disciplinary interests. Back-and-forth manipulations may be a temporary winning strategy for individual factions, but such rule-altering tactics will have negative long-term impacts on the decision process. The opinion of the scientists involved, including emerging young people with future stakes, needs to be safeguarded and respected over organizational priorities and self-interest. Lobbying campaigns with direct lines of communication to national leaders may help jump-start some projects, but in the long run they also erode the administrative chain of command, add unnecessary burdens to top leaders and reduce procedural transparency.

Finally, it may be more effective and inclusive to separate the Chinese Academy of Sciences’ responsibilities of major resource allocation, national facility operation and large project planning and promotion. There will be no perfect solutions. Fortunately, China's investment in basic science research is growing rapidly. Hopefully, with the influx of support and young talents, China will soon spearhead discovery and advancement on many scientific frontiers, including planetary astrophysics. At the same time, the community should think about how to maximize the cost-effectiveness and scientific return of the investment and support of the central government. The ultimate showpieces should be the fruitful discoveries enabled by the mega-facilities rather than the physical shrines themselves. Big and expensive projects are not necessarily the only way to achieve major global impact and breakthroughs.

## A YOUNG FIELD FOR YOUNG RESEARCHERS

***NSR***: You worked as founding director of the Kavli Institute for Astronomy and Astrophysics (KIAA) at Peking University from

2007 to 2011. How do you see the efforts in attracting international talents to work in China?

Collaboration is particularly important in astronomy because modern observers look over a wide range of wavelengths and they need to exchange information to get a complete picture of a given phenomenon.

—Douglas Lin

***Lin***: There are a lot of opportunities for the globalization of frontier science because it is not confined by national boundaries, especially in the field of astronomy where we are all looking at the same sky. KIAA was envisioned to be the ideal platform for cultivating an international atmosphere of openness. But there are also plenty of challenges. One common practice in China is to use certain rigid metrics to judge the performance of an institute, so the institute constantly needs to justify its existence. However, I did not see the need for our institute to compete for the number one spot in China. Instead, I hoped to develop KIAA into an international center of excellence where people from all over the world could get together and discuss ideas; an incubator that motivates innovation and an intellectual forum to inspire bright and promising students to explore in their formative years and to mature into the original thinkers of the future. These lofty goals are quite difficult to achieve, because you can’t simply tell people, ‘Look, I’m investing in a scholarly environment that *may* bear fruit in 20 years’ time’. I believe KIAA is moving in the right direction and it will eventually grow into a world-class institute, where faculty and students are intellectually stimulated, and creativity and interdisciplinary collaboration flourishes in an environment where people enjoy working and living.

***NSR***: Do you think we need to encourage more ‘daring explorers’ to work on hard problems, especially in basic science?

***Lin***: Definitely. If you look at the most important advances in astronomy, many were made serendipitously. For example, when people set out to search for planetary systems that look like ours, the first exoplanet they found turned out to be very different from those in the solar system. However, these serendipitous discoveries were not entirely made by chance. You need facilities, funding, people who are dedicated to exploration, and a little bit of room for out-of-the-box thinking.

I was born one of those Sputnik babies. I got interested in astronomy at age six. I hope today's kids and young people will be inspired by the space programs happening now. In fact, the field of exoplanet research is filled with young people who are imaginative, daring and have very few preconceived constraints on their scientific outlooks. They are very versatile and innovative in utilizing the latest hi-tech inventions and state-of-the-art facilities to gain glimpses into the hitherto-unseen universe. Many have natural talent in designing sophisticated computational algorithms for the mining of well-hidden signals from huge data sets. Some of them also come up with ingenious methods and clever techniques that do not necessarily require huge investments or costly instruments like those I have mentioned.

***NSR***: Would you like to give some advice to young scientists, with examples from your own career path?

***Lin***: I often share with my students four beneficial suggestions with regard to launching a successful scientific career.

First, it is important to follow your heart so that you can embrace challenging goals with a passionate determination and an insatiable curiosity rather than for fame and secure employment. The pursuit of knowledge is a noble motivation in itself.

Second, you may wish to ask, ‘What is the question?’ before ‘What is the answer?’ As a teacher, I prefer to inspire and nourish the potential of original thinkers. Albert Szent-Georgi, who discovered vitamin C, once remarked, ‘Discovery consists of seeing what everybody has seen and thinking what nobody has thought.’ While it is imperative to humbly learn from mentors and peers, you can only explore unchartered waters and advance the frontiers by keeping an open mind and by thinking on your own feet.

**Figure:**
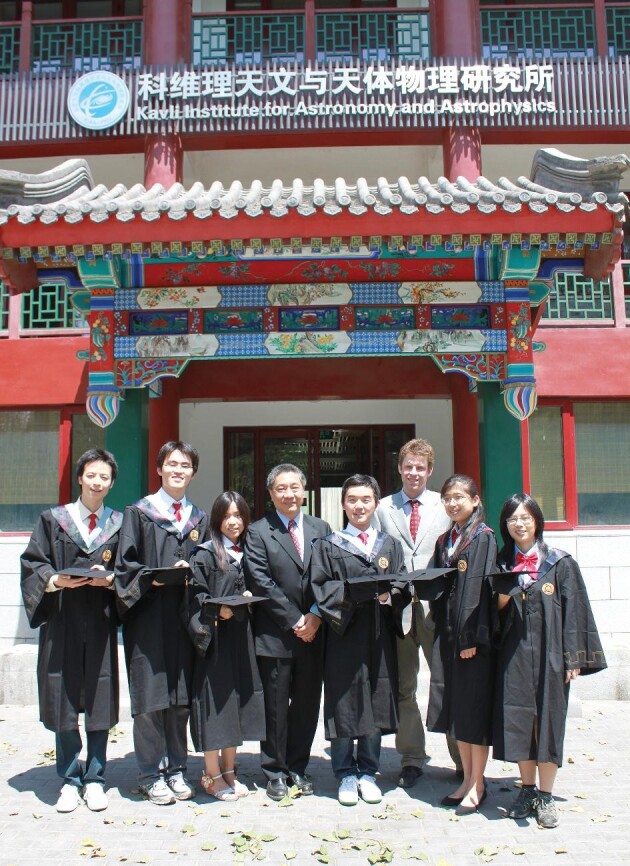
Lin with undergraduate interns in front of the KIAA building in 2008 *(courtesy of Douglas Lin)*.

Third, it is advisable to find and adopt one or more methodologies that are most suitable for you. Some people have special talents in math, some are skillful at experimental techniques, some are gifted in data mining and some are inventive in building instruments to enable others to make discoveries. All this expertise is needed in scientific research. For me, what I enjoy doing the most is deciphering overall connections between complex astrophysical phenomena based on my past exposure and knowledge of competing physical processes. But I don’t have to attempt everything on my own. I admire and respect the complementary skills and expertise of many colleagues and actively initiate numerous fruitful collaborations with my contemporaries and students. Find your natural talent and be confident in yourself while appreciating, learning and benefiting from other people's mastery.

Fourth, since science is a community endeavor, we have a responsibility to effectively disseminate our discoveries. You need to make your findings easily understandable by a large audience so your ideas can become influential. Train yourself to do elevator talks, to successfully convey the essence of your work to informed laymen during a brief ride in an elevator as it ascends from the first to the fourth floor. I find it fulfilling to share my own sense of enthusiasm with people working outside my field, to convey the broad implications of my results and to stoke their intellectual curiosity and inspiration.

## LOOKING FORWARD

***NSR***: Can you summarize your recent research interests and what you’d like to do in the future?

***Lin***: Over the past five years, I have been focusing on the study of gravitational wave events. Recently detected signals come from the fusion of stellar-mass black hole pairs. During their final coalescence, these black holes strongly warp the space-time around them and generate gravitational waves that propagate over great distances. We can spot them with very sensitive detectors on Earth. The question I am most interested in is where these black hole binaries came from and how they merged into more massive entities. A widely adopted theoretical model at the time of discovery was that these black hole pairs are the evolutionary byproducts of binary-star systems. As we gathered more data, this interpretation encountered challenges in explaining the mass range and spin rate of the merging black holes.

In my search for an alternative explanation, I am inclined to link these events with active galactic nuclei that contain accretion disks around some very massive black holes. Based on my previous studies of planet formation and accretion disks, I suggested that gaseous accretion disks around these supermassive black holes in active galactic nuclei can provide an ideal venue for the birth and growth of stellar-mass black holes, their subsequent pairing and eventual coalescence. In this scenario, many problems I had worked on in the context of planet formation turned out to have direct analogous application. So, I work with some valiant young people and encourage them to think out of the box. Along the way, we have discovered some previously neglected physical effects, which may, in turn, be relevant in the analyses of planet formation itself.

During my career as an astrophysicist, rapid advancements in technology have enabled us to gain a global perspective based on the explosively growing multi-wavelength and multi-messenger observational data sets. These fresh clues have ushered in an exciting golden era in astrophysics on many fronts, ranging from exoplanets to astrobiology, black holes, active galactic nuclei and gravitational waves. The intriguing connections and tantalizing complementarities between these physical and conceptual domains, across vastly diverse spatial and temporal scales, have provided a fertile ground for past and potential breakthrough discoveries. I feel very fortunate, privileged and thankful to have

taken part in these stimulating endeavors with the support of the communities and societies in the many countries where I have lived, studied and worked throughout my life. I also envy today's young people who have so many opportunities to explore, contemplate novel ideas, pursue milestone advancements and establish a more comprehensive and accurate understanding of the universe and its sumptuous constituents.

